# Urban Public Health: Is There a Pyramid?

**DOI:** 10.3390/ijerph10020490

**Published:** 2013-01-28

**Authors:** Meirong Su, Bin Chen, Zhifeng Yang, Yanpeng Cai, Jiao Wang

**Affiliations:** 1 State Key Joint Laboratory of Environment Simulation and Pollution Control, School of Environment, No. 19, Xinjiekouwai St., Beijing Normal University, Beijing 100875, China; E-Mails: chenb@bnu.edu.cn (B.C.); zfyang@bnu.edu.cn (Z.Y.); caiyanpeng@iseis.org (Y.C.); 2 Institute for Energy, Environment and Sustainable Communities, University of Regina, 120-2 Research Drive, Regina, Saskatchewan S4S 7H9, Canada; 3 Civil and Environmental Engineering Department, Louisiana State University, Baton Rouge, LA 70803, USA; E-Mail: jwang53@lsu.edu

**Keywords:** urban public health, pyramid structure, development trend, balance, diversity

## Abstract

Early ecologists identified a pyramidal trophic structure in terms of number, biomass and energy transfer. In 1943, the psychologist Maslow put forward a pyramid model to describe layers of human needs. It is indicated that the pyramid principle is universally applicable in natural, humanistic and social disciplines. Here, we report that a pyramid structure also exists in urban public health (UPH). Based on 18 indicators, the UPH states of four cities (Beijing, Tokyo, New York, and London) are compared from the point of view of five aspects, namely physical health, living conditions, social security, environmental quality, and education and culture. A pyramid structure was found in each city when focusing on 2000–2009 data. The pyramid of Beijing is relatively similar to that of Tokyo, and the pyramids of New York and London are similar to each other. A general development trend in UPH is proposed and represented by different pyramid modes. As a basic conjecture, the UPH pyramid model can be verified and developed with data of more cities over a longer period, and be used to promote healthy urban development.

## 1. Introduction

Urban ecosystem health is essential for regional, national and even global development [1,2]. It emphasizes ecosystem renewability and the ability to provide services for human beings [3,4,5]. Urban public health (UPH), an important component of urban ecosystem health, is a global health issue and receives a great deal of attention [6]. With the goal of preventing disease, prolonging life and promoting health and efficiency [7], UPH emphasizes factors of health services and access [8,9], economic condition [10,11], standards of living [12,13], education [10,12], environment [14,15,16,17], and social stability [12,18,19]. These factors are of more concern and are easily accepted by the public and managers compared with the concept of urban ecosystem health.

It is generally accepted that these factors are all important for UPH. But there remain some really interesting questions, e.g., what is the relationship among these factors? Is there a balance among them? How will the relationship mode change with time? Will the relationships differ from one city to another?

As is widely known, early ecologists have identified a pyramidal trophic structure in terms of number, biomass and energy transfer [20,21,22]. And the psychologist Maslow put forward a pyramid model to describe layers of human needs [23]. It is indicated that the pyramid principle is universally applicable in natural, humanistic and social disciplines. Focusing on above-mentioned questions, a pyramid structure is also found in UPH. And a general development trend in UPH is proposed in this paper. The relationship among UPH pyramid, ecological pyramid and Maslow’s pyramid is also further discussed.

## 2. Methods

### 2.1. Assessment Indicators of UPH

Based on the above factors of concern (*i.e*., health services and access, economic conditions, standards of living, education, environment, and social stability) and the main function of cities for the public (a place to live, study, work and develop), the status of UPH is described by five factors, including physical health, living conditions, social security, environmental quality, and education and culture. According to related indicators of urban ecosystem health and public health [3,4,5,6,7,8,9,10,11,12,13,14,15,16,17,18,19], the correlation analysis of different indicators, and data availability, the five UPH factors are finally measured by selected eighteen indicators, as shown in Table 1.

**Table 1:** Urban public health indicators.

Factors	Indicators	Unit
Physical health	Infant mortality rate	‰
	Life expectancy	years old
	Number of doctors per 1,000 persons	-
	Number of hospital beds per 1,000 persons	-
Living conditions	*Per capita* GDP	US $
	Angel’s coefficient	-
	*Per capita* living space	m^2^
	Average commuting time	min
Social security	Unemployment rate	%
	Average daily crime incidents	-
	Traffic deaths per 10,000 vehicles	-
Environmental quality	COD emission intensity per unit GDP	kg/10^4^ US $
	Daily average SO_2 _concentration	mg/m^3^
	Daily average NO_2 _concentration	mg/m^3^
	*Per capita* public green areas	m^2^
Education and culture	Gross enrolment rate in tertiary education	%
	Internet penetration rate	%
	Number of libraries per million persons	-

### 2.2. Data Collection

The required data of the eighteen indicators for the 2000–2009 period was collected from various yearbooks and websites such as the Organization for Economic Co-operation and Development, World Bank, and United Nations Educational, Scientific and Cultural Organization. Most of data was collected at the city scale, while a small number was represented by national data, such as number of hospital beds per 1,000 persons, gross enrolment rate in tertiary education, and internet penetration rate.

### 2.3. Weighted Sum Model

To quantify the five UPH factors, the weighted sum method is used. Indicator normalization is first performed via Equations (1) and (2):

*S_i_* = *X_i_*/*X_max_* (1)

*S_i_* = *X_min_*/*X_i_* (2)

where Equation (1) is applicable for positive indicators that express a better health level with a larger indicator value, while Equation (2) is applicable for negative indicators. *S_i_* is the standardized value of the *i*th indicator, *X_i_* is the original value of the *i*th indicator, and *X_max_* and *X_min_* are the maximum and minimum values of the *i*th indicator.

Then, considering the indicators’ weights, the quantitative result for each UPH factor is obtained by Equation (3):

*V_j_* = ∑*S_i_*×*ω_i_* (3)

where *V_j_* is the value of the *j*th factor, and *ω_i_* is the weight of the *i*th indicator relative to the *j*th factor. Here, fair weight is adopted to simplify the problem. For example, the weights of four indictors under the physical health factor are all one quarter, and the weights of three indicators under social security are all one third.

### 2.4. Study Sites

Beijing, which aims to become global city, and three acknowledged global cities, Tokyo, New York and London, were chosen as the study sites. Beijing developed rapidly after the policy of reform and opening was implemented in 1978. Tokyo experienced rapid development in the 1950s. New York became a global economic center in 1825 and developed towards being a global metropolis since the early 20th century. London experienced rapid socio-economic development in the 18th century, especially following the industrial revolution.

## 3. Results

### 3.1. General UPH States of Four Cities

**Figure 1:**
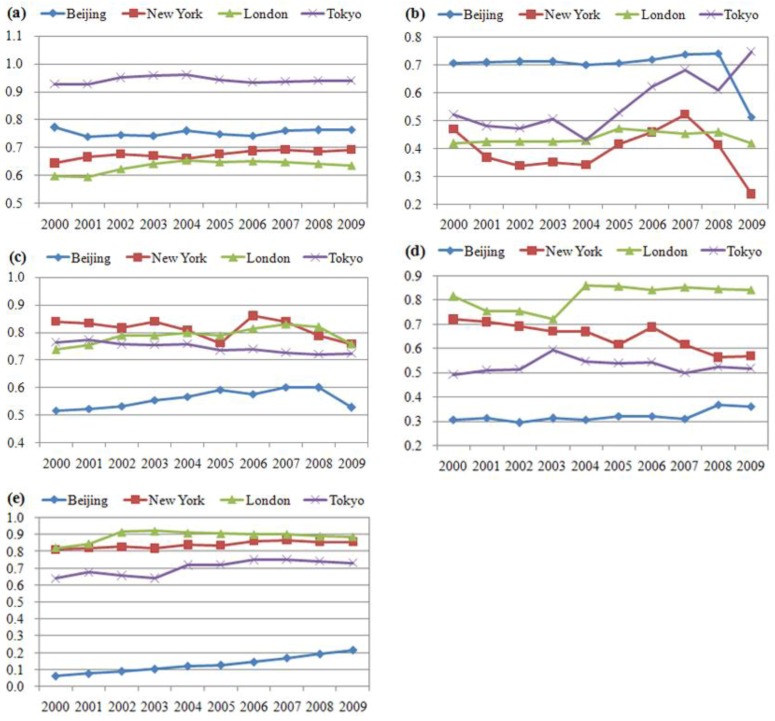
Relative situation of five UPH factors for four cities in 2000–2009. (**a**) physical health. (**b**) social security. (**c**) living condition. (**d**) environmental quality. (**e**) education and culture.

After collecting data of eighteen 2000–2009 indicators, the estimated values of five UPH factors for Tokyo, New York and London were calculated based on weighted sum model. The comparative results are displayed in Figure 1, in which the relative position of each city during 2000–2009 is roughly shown with respect to each UPH factor. 

The New York and London values are always similar, although with different changing trends for most factors. The Tokyo values are usually adjacent to those of New York and London, while the changing trends for most factors are basically similar to those of Beijing. 

### 3.2. UPH Pyramid of Each City

**Figure 2:**
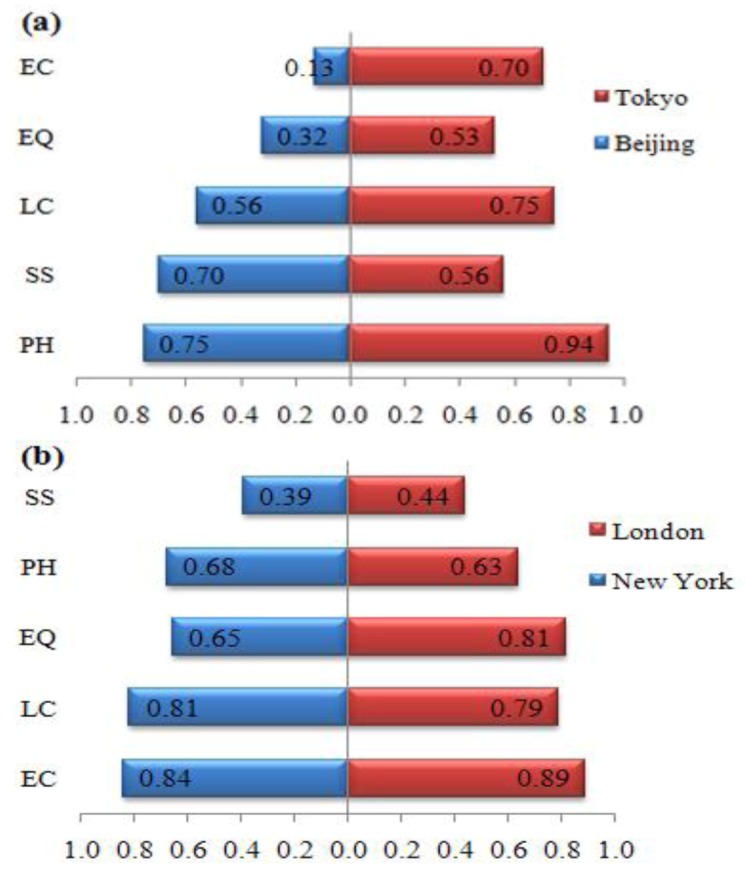
UPH pyramid structures for four cities in 2000–2009. (**a**) Tokyo-Beijing. (**b**) New York-London. The mean value during 2000–2009 is adopted here to compare the five UPH factors for each city. PH, physical health; SS, social security; LC, living condition; EQ, environmental quality; EC, education and culture.

Based on these 2000–2009 values, a comparison among the five UPH factors for each city was further conducted, through which the relative development strength of each factor can be observed. A pyramid structure among different UPH factors was found for each city, in which the pyramid of Beijing is relatively similar to that of Tokyo, and the pyramid of New York is similar to that of London, as indicated in Figure 2.

### 3.3. UPH Pyramid Development Trend

**Figure 3:**
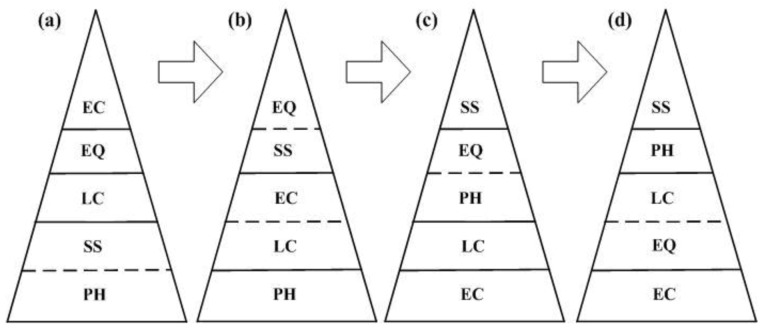
UPH development trend. (**a**) Beijing mode. (**b**) Tokyo mode. (**c**) New York mode. (**d**) London mode. PH, physical health; SS, social security; LC, living condition; EQ, environmental quality; EC, education and culture. The factors with larger calculated values locate at the bottom of the pyramid, meaning that these factors’ situations are better than those of other cities. Factors with smaller values locate at the top, meaning that the situations are weaker. The values of two factors separated by a dotted line are very close to each other.

Considering the development history of the four cities, especially the age at which they experienced socio-economic acceleration after certain events such as the industrial revolution and political and economic innovation, the global UPH development trend is proposed, as shown in Figure 3. Here, the development trend is represented by different cities with different developmental ages, rather than by different stages of the same city.

With respect to the physical health factor, its importance gradually declines when a certain standard is reached. For the social security factor, the situation becomes worse when compared with other factors, *i.e.*, it moves from the bottom of the pyramid to the top. The living condition factor is always important and locates at the bottom or middle of the pyramid. In terms of the environmental quality factor, it worsens before improving, which is somewhat consistent with the Environmental Kuznets Curve [24]. With regard to the education and culture factor, it gradually becomes highly valued in the development process.

## 4. Discussion

In terms of the ecological pyramid, each component is equally vital for maintaining a holistic ecological balance, although the components locate at different layers of the pyramid. Similarly, each UPH factor is equally important, although the factors locate at different layers. In a natural ecosystem, the pyramid structure is relatively fixed, and the energy transfers along the food chain, *i.e.*, from producer to junior consumer and then to senior consumer. But the UPH situation is quite different. In the human-dominant urban ecosystem, human value standards and policy decisions differ with actual present conditions and may change with time. Therefore, there are different pyramid modes for different cities as well as the same city in different periods. It should be emphasized that it is always a pyramid rather than some other shape such as a rectangle or circle, meaning that there is a balance between different factors, *i.e.*, some factors falling and the others rising at the same time. This kind of balanced and dialectical principle is behind the continuous driving force of urban development.

According to Maslow’s human needs pyramid, the structure is fixed, *i.e.*, basic needs such as food, sleep and safety locate at the bottom of the pyramid, needs for social life and esteem at the middle, and need for self-actualization at the top. It is a little similar to the UPH mode in Figure 3(a), in which physical health and social security, ensuring human survival, locate at the bottom of the pyramid; living conditions, enabling work and respect, at the middle; and education and culture, assisting self-realization, at the top. However, the UPH pyramid structure will change into other modes, such as those shown in Figure 3(b–d). Even within the same city, the UPH pyramid structure changes with time. In recent research, Diener found that people can experience good social relationships and self-realization even though their basic needs are not fully satisfied [25]. We assume that Maslow’s pyramid of human needs may also have different modes with social development and human aging.

In terms of different UPH modes, we cannot arbitrarily say which one is relatively more high-ranking than others, at least not with the present limited data. On the contrary, diversity is potentially a good choice for continuously promoting global development with a holistic perspective. With enough data of more global cities, different UPH pyramid modes can be further verified. Potentially, more typical modes will be found, allowing the UPH pyramid development trend to be described in more detail. With more data of more cities over a longer period, the usual development trends with time for different cities can be observed and compared. This means that for each city, different UPH pyramid modes will happen one after another, showing the UPH development trend over time. Similar temporal development trends for different cities might be found. The pyramid modes listed in this paper provide a baseline UPH development trend, on which more pyramid modes over a longer time series can be further studied.

It is undoubted that indicator selection directly impacts on the final results of UPH. Although various factors including concerns of UPH, function of cities for the public, related existent indicators of urban ecosystem health and public health, and data availability, are all considered, more amendment is still necessary in the future to obtain a more scientific result. Especially for UPH that closely linked to human needs and actual management, certain supplements and modifications to the present indicators should be carried out according to the academic development in related subjects and change of human values. Meanwhile, how to confirm the indicator weights is always an open question [26] since each method has its own advantages and disadvantages. To simplify the problem of weight confirmation, fair weight is adopted in this paper. Further attempt is deserved to incorporate different methods to define indicator weights. However, it is still predicted that pyramid structure can be found in UPH although with different indicators and weights.

## 5. Conclusions

We reported that there exists pyramid structure in UPH. Although the concrete pyramid modes differ from some cities to others, it is always a pyramid rather than some other shape. It means that there is a balance between different factors of UPH. We also preliminarily proposed the global UPH development trend, which was represented by different pyramid modes. And different cities indicated diversity at a certain point in time with respect to pyramid modes of UPH. This kind of balance and diversity is potentially a good choice for continuously promoting global healthy urban development with a holistic perspective.

## References

[1] Huang S.L. (1998). Urban ecosystems, energetic hierarchies, and ecological economics of Taipei metropolis. J. Environ. Manag..

[2] Su M.R., Yang Z.F., Chen B. (2010). Relative urban ecosystem health assessment: A method integrating comprehensive evaluation and detailed analysis. Ecohealth.

[3] Costanza R., Costanza R., Norton B.G., Haskell B.D. (1992). Toward an operational definition of ecosystem health. Ecosystem Health: New Goals for Environmental Management.

[4] Rapport D.J., Gaudet C., Karr J.R., Baron J.S., Bohlen C., Jackson W., Jones B., Naiman R.J., Norton B., Pollock M.M. (1998). Evaluating landscape health: Integrating societal goals and biophysical process. J. Environ. Manag..

[5] Su M.R., Fath B.D., Yang Z.F. (2010). Urban ecosystem health assessment: A review. Sci. Total Environ..

[6] Khan M.H. (2012). Urban health in megacities of developing countries. Publ. Health Forum.

[7] Winslow C.E.A. (1920). The untilled fields of public health. Science.

[8] Garcia-Lacalle J., Martin E. (2010). Rural *vs.* urban hospital performance in a ‘competitive’ public health service. Soc. Sci. Med..

[9] Zhao Y., Cui S., Yang J., Wang W., Guo A., Liu Y., Liang W. (2011). Basic public health services delivered in an urban community: A qualitative study. Publ. Health.

[10] Dahlgren G., Whitehead M. (1991). Policies and Strategies to Promote Social Equity in Health. http://www.framtidsstudier.se/wp-content/uploads/2011/01/20080109110739filmZ8UVQv2wQFShMRF6cuT.pdf.

[11] Liu S., Griffiths S.M. (2011). From economic development to public health improvement: China faces equity challenges. Publ. Health.

[12] World Health Organization (1986). The Ottawa Charter for Health Promotion. http://www.who.int/healthpromotion/conferences/previous/ottawa/en/.

[13] Whiteis D.G. (1998). Third world medicine in first world cities: Capital accumulation, uneven development and public health. Soc. Sci. Med..

[14] St Leger L. (2003). Health and nature—New challenges for health promotion. Health Promot. Int..

[15] Brown T., Bell M. (2007). Off the couch and on the move: Global public health and the medicalisation of nature. Soc. Sci. Med..

[16] Su M.R., Yang Z.F., Chen B., Ulgiati S. (2009). Urban ecosystem health assessment based on emergy and set pair analysis—A comparative study of typical Chinese cities. Ecol. Model..

[17] Wu Z., Li Z., Chen G.Q. (2011). Multi-scale analysis for environmental dispersion in wetland flow. Commun. Nonlinear Sci. Numer. Simul..

[18] Harpham T. (2009). Urban health in developing countries: What do we know and where do we go?. Health Place.

[19] Wang J., Su M.R., Chen B., Chen S.Q., Liang C. (2011). A comparative study of Beijing and three global cities: A perspective on urban livability. Front. Earth Sci..

[20] Elton C.S., Elton C.S. (1927). The animal community. Animal Ecology.

[21] Davis M.A., Thompson K., Grime J.P., Charles S. (2001). Elton and the dissociation of invasion ecology from the rest of ecology. Divers. Distrib..

[22] Odum E.P., Barrett G.W. (2004). Fundamentals of Ecology.

[23] Maslow A.H. (1943). A theory of human motivation. Psychol. Rev..

[24] Shafik N. (1994). Economic development and environmental quality: An econometric analysis. Oxf. Econ. Paper..

[25] Diener E., Ng W., Harter J., Arora R. (2010). Wealth and happiness across the world: Material prosperity predicts life evaluation, whereas psychosocial prosperity predicts positive feeling. J. Pers. Soc. Psychol..

[26] Su M.R., Fath B.D. (2012). Spatial distribution of urban ecosystem health in Guangzhou, China. Ecol. Indicat..

